# Hypomorphic biallelic *LIFR* variants define a multisystem disorder with survival beyond infancy and hyperinflammatory vulnerability

**DOI:** 10.3389/fimmu.2026.1927542

**Published:** 2026-10-19

**Authors:** Madeleine Schmidt, Puyan Rafii, Aisha AlShamsi, Giovanni Zifarelli, Maxi Leona Wiese, Joachim David Steiner, Sarah Kreuz, Robert Walter Körner, Clara Velmans, Christian Netzer, Oliver Semler, Majid Alfadhel, Hiba Abu Khalil, Ezgi Susam, Sujatha Jagadeesh, Beena Suresh, Nicolai Kohlschmidt, Philip M. Boone, Sheela Nampoothiri, Radhakrishnan Periyasamy, Katta M. Girisha, Andreas Hahn, Adela Chirita-Emandi, Raphael Hirtz, Kai O. Hensel, Peter Bauer, Konstantin L. Makridis, Angela M. Kaindl, Ilja Dubinski, Jürgen-Christoph von Kleist-Retzow, Gregor Dückers, Reem Elfeky, Maaike Kusters, Tim Niehues, Vivien Béziat, Heinz Jungbluth, Adam Antebi, Jürgen Scheller, Hormos S. Dafsari

**Affiliations:** 1 Department of Pediatrics, Faculty of Medicine and University Hospital Cologne, University of Cologne, Cologne, Germany; 2 Institute of Biochemistry and Molecular Biology II, Medical Faculty and University Hospital Düsseldorf, Heinrich Heine University Düsseldorf, Düsseldorf, Germany; 3 Division of Genetics and Metabolic, Department of Paediatrics, Tawam Hospital, Al Ain, United Arab Emirates; 4 CENTOGENE GmbH, Rostock, Germany; 5 Max-Planck-Institute for Biology of Ageing and Cologne Excellence Cluster on Cellular Stress Responses in Aging Associated Diseases, Cologne, Germany; 6 Department II of Internal Medicine and Center for Molecular Medicine Cologne, University of Cologne, Cologne, Germany; 7 Center of Excellence in Nephrology, University Hospitals Aachen, Cologne, Dusseldorf, North Rhine-Westphalia, Germany; 8 Institute of Human Genetics, Faculty of Medicine and University Hospital, University of Cologne, Cologne, Germany; 9 Medical Genomic Research Department, King Abdullah International Medical Research Center (KAIMRC), King Saud Bin Abdulaziz University for Health Sciences (KSAU-HS), Ministry of National Guard Health Affairs (MNG-HA), Riyadh, Saudi Arabia; 10 Genetics and Precision Medicine Department (GPM), King Abdullah Specialized Children’s Hospital (KASCH), King Abdulaziz Medical City, Ministry of National Guard Health Affairs (MNG-HA), Riyadh, Saudi Arabia; 11 Division of Medical Genetics, Sakarya Research and Training Hospital, Sakarya, Türkiye; 12 Department of Clinical Genetics & Genetic Counseling Mediscan Systems, Chennai, India; 13 Institute of Clinical Genetics and Tumor Genetics, Medizinisches Versorgungszentrum (MVZ), Bonn, Germany; 14 Division of Genetics and Genomics, Boston Children’s Hospital, Boston, MA, United States; 15 Department of Pediatric Genetics, Amrita Institute of Medical Sciences & Research Centre, Kochi, India; 16 Department of Medical Genetics, Kasturba Medical College, Manipal, Manipal Academy of Higher Education, Manipal, India and Suma Genomics Private Limited, Manipal, India; 17 Department of Child Neurology, University Hospital, Gießen, Germany; 18 Genetics Department, Center for Genomic Medicine, University of Medicine and Pharmacy “Victor Babes”, Timisoara, Romania; 19 Witten/Herdecke University, Department of Pediatrics, Faculty of Health, Helios University Hospital Wuppertal, Children’s Hospital, Wuppertal, Germany; 20 Department of Pediatric Neurology, Charité - Universitätsmedizin Berlin, Berlin, Germany; 21 Center for Chronically Sick Children, Charité - Universitätsmedizin Berlin, Berlin, Germany; 22 Institute of Cell- and Neurobiology, Charité – Universitätsmedizin Berlin, Berlin, Germany; 23 Section Central Nervous System (CNS) Development and Neurologic Disease, Partner Site Berlin, German Center for Child and Adolescent Health (Deutsches Zentrum für Kinder- und Jugendgesundheit), Berlin, Germany; 24 Dr von Hauner’s Children’s Hospital, Ludwig-Maximilians-Universität München Munich, Munich, Germany; 25 Department of Pediatrics, Helios Klinikum, Krefeld, Germany; 26 Department of Immunology, Great Ormond Street Hospital for Children NHS Foundation Trust, London, United Kingdom; 27 HMU Health and Medical University, Campus Düsseldorf/Krefeld, Düsseldorf, Germany; 28 Université Paris Cité, Institut Imagine, Laboratory of human genetics of infectious diseases, Necker branch, INSERM UMR 1163, Paris, France; 29 Department of Paediatric Neurology, Evelina London Children’s Hospital, London, United Kingdom; 30 Randall Centre for Cell and Molecular Biophysics, Muscle Signalling Section, Faculty of Life Sciences and Medicine, King’s College London, London, United Kingdom; 31 Center for Rare Diseases, Faculty of Medicine and University Hospital Cologne, University of Cologne, Cologne, Germany; 32 Center for Molecular Medicine Cologne (CMMC), Faculty of Medicine and University Hospital Cologne, University of Cologne, Cologne, Germany

**Keywords:** ERK signaling, genotype-phenotype correlation, inborn errors of immunity, intracellular trafficking, JAK/STAT 3 signaling, LIFR deficiency, MIS-C, neurodevelopmental disorders

## Abstract

**Background:**

The leukemia inhibitory factor receptor (LIFR) is a key signaling receptor within the IL-6 cytokine-family, and its integrity is essential for STAT3 and ERK activation in developmental and immune pathways. Recessive, predominantly truncating variants in *LIFR* cause Stüve-Wiedemann syndrome (SWS), historically a neonatal-lethal skeletal dysplasia. As more individuals survive beyond infancy, the clinical spectrum and its biological basis remain incompletely defined.

**Methods:**

We describe the clinical heterogeneity with considerable survival beyond infancy and phenotypic expansions in neurodevelopment and immune dysregulation and determine how missense variants alter cytokine and receptor trafficking. To define the expanded disease spectrum and underlying mechanisms, we assembled an international cohort of 22 previously unreported individuals with biallelic *LIFR* variants, combined with functional characterization of missense alleles using cellular receptor abundance and STAT/ERK signaling assays, and proteomic profiling of patient-derived bronchoalveolar lavage (BAL) cells.

**Results:**

We identified a markedly expanded phenotype among survivors beyond infancy, including neurodevelopmental disorders, allergy, and hyperinflammatory courses, including COVID-19–associated multisystem inflammatory syndrome in children (MIS-C). In cellular assays, the hypomorphic missense variant p.Cys270Gly showed reduced cell-surface LIF-receptor abundance, STAT/ERK phosphorylation and cellular proliferation upon LIF stimulation. Analysis of additional missense variants revealed functional domain-specific effects: N-terminal extracellular variants retained partial activity, whereas C-terminal cytoplasmic variants showed near-complete loss-of-function. Patient bronchoalveolar-cell proteomics identified dysregulations in endolysosomal recycling of defective LIF-receptor.

**Discussion:**

Our cohort’s findings may position LIFR deficiency as a survivable multisystem disorder with relevant neurological and autoimmune-inflammatory vulnerability. Variant-level functional stratification and proteomic signatures provide a coherent biological framework to support risk assessment and long-term clinical management in affected individuals.

## Introduction

Stüve-Wiedemann syndrome (SWS) was first described in two patients (1) with characteristic features of bone abnormalities, short stature, episodic hyperthermia, respiratory distress, and feeding difficulties. Biallelic variants in the leukemia inhibiting factor receptor gene (*LIFR*) were identified as a cause for Stüve-Wiedemann syndrome type 1 (2) More recently, biallelic variants in the interleukin-6 cytokine family signal transducer gene (*IL6ST*/gp130) were associated with a similar phenotype named Stüve-Wiedemann syndrome type 2 (3). Loss-of-function variants in any of these genes lead to a perturbation in interleukin-6 signal transduction that may affect intracellular Janus kinase (JAK) phosphorylation as well as activation and nuclear translocation of signal transducers and activators of transcription protein (STAT) (4). Previous basic science research indicated the role of LIFR in the function and development of post-mitotic plasmacytoid dendritic cells (5) as well as matured cortical neurons (6), both found in post-mitotic tissue. Recent evidence identified endosomal internalization and endolysosomal trafficking of LIFR for subsequent degradation (7, 8).

Genotype-phenotype correlations for pathogenic *IL6ST* variants have shown location-dependent perturbations in domain function and, ultimately, defective cytokine response (4, 9, 10). While patients with *LIFR*-related SWS have predominantly been reported with infantile death, there is a lack of detailed phenotypic characterization of the multi-system involvement in this syndromic immunodeficiency, particularly in “long-term survivors” after infancy (11–13).

In this study, we set out to i) report 22 novel patients with a spectrum of *LIFR* variants and variable phenotypic progression, ii) examine the *LIFR* genotype-phenotype spectrum with hypomorphic patient alleles in cell assays to examine domain-specific loss-of-function, and iii) investigate dysregulated endolyosomal trafficking downstream of defective LIF-receptor which is particularly relevant for the phenotype in postmitotic tissues including skeleton, neurons and immune cells.

## Materials and methods

### Patient characterization and genotype-phenotype correlation analyses

The patients were recruited by multi-center study sites via GeneMatcher, ERN ITHACA or ESNEK, each with regional IRB approval (20-1711, Medical Faculty of University Cologne, Germany) (14). To supplement our analyses on allelism, we conducted a genotype-phenotype correlation study in 74 patients with variants in *LIFR* of which n=22 are novel patients collected in our cohort and identified domain-specific phenotype clustering (**Supplementary Material**) (2, 9, 15–37).

### Patient recruitment

We examined publications in PubMed for patients with *LIFR-*related SWS from 01.01.2010 to 01.09.2023 and identified 24 publications with 52 patients (2, 9, 15–33, 35–37).

We re-evaluated cases of cohorts with n>5 cases regarding the core features immunodeficiency, skeletal dysplasia, and neurological disorders (neurodevelopmental disorders, muscular hypotonia, neurodegenerative courses). For all indicated percentages below, we identified the reported phenotypes from published patients and novel patients from our cohort. Unreported phenotypes in patients were designated as ‘not defined’ (ND). The reported percentages below present the percentage of manifested phenotypes from the total number of patients.

### Cloning

The hLIFR (P42702) in pcDNA3.1 vector was used as a template for side directed mutagenesis using the primer: C270G fw 5’ CAT AAC ATT TGG TTG TGT GAG TCA AG, C270G rev 5’ TCT GAG CCT ACA AGT ATC AC. Gene coding for wildtype and LIFR variants p.Asn128Lys, p.Pro210Cys, p.Cys270Gly, p.Thr646Asn, p.Asn1096Lys, and p.Tyr697Ter were then inserted into pMOWS retroviral vector including hygromycin B resistance.

### Cells, reagents and recombinant proteins

The generation of Ba/F3-gp130 cells was described elsewhere (38). The packaging cell line Phoenix-Eco was received from Ursula Klingmüller (DKFZ, Heidelberg, Germany). HEK293T (ACC-635) cells were purchased from the Leibniz Institute DSMZ-German Collection of Microorganisms and Cell Culture (Braunschweig, Germany). All cells were grown at 37 °C with 5% CO_2_ in a water-saturated atmosphere in Dulbecco`s modified Eagle`s medium (DMEM) high-glucose culture medium (GIBCO^®^, Life Technologies, Darmstadt, Germany) with 10% fetal calf serum (GIBCO^®^, Life Technologies) and 60 mg/l penicillin and 100 mg/l streptomycin (Genaxxon Bioscience GmbH, Ulm, Germany). Murine Ba/F3-gp130 cells were obtained from Immunex (Seattle, WA, USA) and grown in the presence of the fusion protein Hyper-IL-6 (HIL-6). 0.2% (10ng/ml) conditioned medium from a stable clone of CHO-K1 cells secreting HIL-6 in the supernatant (39). HIL-11-Ts was expressed and purified as reported elsewhere (40). Recombinant human LIF (catalog no. 7734-LF) was purchased from R&D Systems (Minneapolis, MN, USA).

### Western blotting

50 µg total protein were loaded per lane and separated by SDS-PAGE under reducing conditions and transferred to a nitrocellulose membrane (Amersham Protan; Cytiva; LC, United Kingdom; catalog no. 10600016). Blocking of membrane was performed with blocking buffer (Intercept^®^ Blocking Buffer; LI-COR; USA; catalog no. 927-60001) diluted 1:3 in TBS (10 mM Tris-HCl pH 7.6, 150 mM NaCl) for 1 h. Primary antibodies (STAT1; catalog no. 9172; phosphor-STAT1; catalog no. 9167; Phospho-STAT3; Tyr-705; D3A7; catalog no. 9145; STAT3; 124H6; catalog no. 9139; ERK1/2; catalog no. 9102; pospho-ERK1/2; catalog no. 4370, Cell Signaling Technology, USA) and (LIFR; catalog no. BAF249, R&D Systems, USA) were diluted 1:1,000 in blocking buffer containing 0.2% Tween-20 (Sigma-Aldrich; USA; catalog no. P1379-1L) for at least 90 min at ambient temperature or overnight at 4 °C. Membranes were washed with TBS-T (0.1% Tween-20) and then incubated with secondary fluorophore-conjugated antibodies 1:10,000 (IRDye^®^ 800CW Donkey anti-Rabbit; catalog no. 926-32213, IRDye^®^ 680RD Donkey anti-Mouse; catalog no. 926–68072 and Donkey anti-Goat; catalog no. 926-32214, LI-COR; USA) for 1 h. Signal detection was achieved using LI-COR Odyssey; USA; Model 2800). Secondary antibodies were detected simultaneously on different channels. Data analysis was conducted using Image Studio Lite 5.2.

### Cell viability assay

Ba/F3-gp130 cells were washed three times with PBS to remove cytokines from the medium. Cells were suspended in DMEM containing 10% fetal calf serum, 60 mg/l penicillin and 100 mg/ml streptomycin at a density of 5x10^4^ cells/ml. Cells were cultured for three days in a volume of 100 µl with or without cytokines or inhibitor in the indicated concentrations. The CellTiter Blue Viability Assay (Promega, Karlsruhe, Germany) was used to determine the approximate number of viable cells by measuring the fluorescence (Λ_ex_560 nm/Λ_em_590 nm) using the Infinite M200 Pro plate reader (Tecan, Crailsheim, Germany). After adding 20 µl/well of CellTiter Blue reagent (time point 0), fluorescence was measured after 60 minutes every 20 min for up to two hours. For each condition of an experiment, three wells were measured. All values were normalized by subtracting time point 0 values from the final measurement.

### Transfection and transduction of cells

Ba/F3-gp130 cell lines were retrovirally transduced with the pMOWS expression plasmids as described previously (41). Transduced cells were grown in DMEM medium as described above supplemented with 10 ng/ml HIL-6. Selection of transduced Ba/F3-gp130 cells was performed with hygromycin B (1 mg/ml) (Carl Roth, Karlsruhe, Germany) for at least 2 weeks. Afterwards, the generated Ba/F3-gp130 cell lines were analyzed for receptor cell surface expression via flow cytometry. HEK293T cells were transfected by 7.5 µg pDNA with 15 µl TurboFect (Thermo Fisher, USA, catalog no. R0532) and incubated for 48 h before cell lysis and Western Blotting.

### Stimulation assay

Ba/F3-gp130 cell lines were washed three times with PBS to remove cytokines and starved in serum-free DMEM for 3h. Cells were stimulated for 15 min with LIF (10 ng/ml) or HIL-11-Ts (50 ng/ml), harvested, frozen in liquid nitrogen and then lysed. Cells were lysed for 45 min with buffer containing 10mM Tris-HCl, pH 7.5, 150mM NaCl, 0.5mM MgCl_2_, 1mM Na_3_VO_4_ and a complete EDTA-free protease inhibitor mixture tablet (Roche Diagnostics, Mannheim, Germany). Protein concentration was determined by a BCA protein assay (Thermo Fisher Scientific) according to the manufacturer’s instruction. Receptor expression or pathway activation was then analyzed by Western Blotting which is described in detail in the **Supplementary Material**.

### Cell surface detection of cytokine receptors via flow cytometry

Cell surface expression of stably transfected Ba/F3-gp130 cell lines was detected by specific antibodies. 5×10^5^ cells were washed in FACS buffer (PBS, 1% BSA) and then incubated in 50 µl of FACS buffer containing the indicated specific primary antibody (anti-LIFR; 1:20 diluted; catalog no. BAF249, R&D Systems; USA). After incubation of 1 h at room temperature, cells were washed and resuspended in 50 µl of FACS buffer containing secondary antibody (NothernLights 493–conjugated anti-goat IgG 1:200; catalog no. NL003, R&D Systems; USA) and incubated for 30 min at room temperature. Cells were washed and resuspended in 500 µl of FACS buffer and analyzed by flow cytometry (BD FACSCanto II flow cytometer using the FACSDiva software, BD Biosciences). Data analysis was conducted using FlowJo Version 10 (Tree Star Inc, US).

Please see **Supplementary Material** for detailed methods on cloning, cells, reagents and recominant proteins, cell viability assay, and transfection and transduction of cells.

### Bronchoalveolar cell sampling and LC-MS/MS proteomics

We conducted LC-MS/MS proteomics on bronchoalveolar cells from a patient with LIFR:p.Cys270Gly (patient 1) and a healthy sex- and age-matched control subject, both samples obtained by flexible bronchoscopy. The bronchoscope was meticulously wedged in the lingula, and a total of four 20 ml aliquots of normal saline, prewarmed to 37 °C, were sequentially instilled and manually aspirated into the same syringe until no further fluid was retrieved. Total recovery rates approximated 70% in both patients. The first aliquot was used for microbiological analysis while the remaining portions were pooled for subsequent investigations. The lavage fluid was promptly transported to the laboratory at room temperature where it was centrifuged down with 400 rpm over 10 min at 4 °C for decanting of supernatant over three times. The cells were then frozen in -70 °C until further use.

We washed bronchoalveolar cells with PBS for five times. Lysis buffer (6 M guanidinium chloride, 2.5 mM TCEP (tris(2-carboxyethyl) phosphine), 10 mM CAA (chloroacetamide), 100 mM Tris-HCl) was added to each sample. Samples were heated at 95 °C for 5–10 min, lysed with the Bioruptor (30-s sonication, 30-s breaks, 10 cycles, 2–3 repeats) and centrifuged at 20,000g for 20 min. Then, 2 µL of the protein supernatant was diluted at least 10 times with 20 mM Tris to 20 µl to reduce the concentration of GuCl to lower than 0.6 M. Protein concentration was then measured by Nano Drop. Samples were then diluted 10 times and digested with trypsin (1:50, Promega, Mass Spec Grade) overnight at 37 °C. The digestion was stopped by adding 50% of FA to 1% (final concentration). Peptides were separated on a 25-cm, 75-μm internal diameter PicoFrit analytical column (New Objective) packed with 1.9 μm ReproSil-Pur 120 C18-AQ medium (Dr. Maisch) using an EASY-nLC 1000 (Thermo Fisher Scientific). The column was maintained at 50 °C. Buffer A and B were 0.1% formic acid in water and 0.1% formic acid in 80% acetonitrile. Peptides were separated on a segmented gradient from 6% to 31% buffer B for 120 min and from 31% to 50% buffer B for 10 min at 200 nL min–1. Eluting peptides were analyzed on a QExactive HF mass spectrometer (Thermo Fisher Scientific). Peptide precursor m/z measurements were carried out at 60,000 resolution in the 300 to 1,800 m/z range. The top 10 most intense precursors with charge state from 2 to 7 only were selected for HCD fragmentation using 25% normalized collision energy. The m/z values of the peptide fragments were measured at a resolution of 30,000 using a minimum AGC target of 8 × 103 and 55-ms maximum injection time. Upon fragmentation, precursors were put on a dynamic exclusion list for 45 s. The raw data were analyzed with MaxQuant version 1.5.2.8 using the integrated Andromeda search engine (42). Peptide fragmentation spectra were searched against the canonical and isoform sequences of the H. sapiens and M. musculus reference proteomes. The digestion parameters were set to ‘specific’ and ‘Trypsin/P,’ The minimum number of peptides and razor peptides for protein identification was 1; the minimum number of unique peptides was 0. Protein identification was performed at a peptide spectrum matches and protein false-discovery rate of 0.01. The ‘second peptide’ option was on. Successful identifications were transferred between the different raw files using the ‘Match between runs’ option. Label-free quantification (LFQ) was performed using an LFQ minimum ratio count of 2. LFQ intensities were filtered for at least four valid values in at least one group and imputed from a normal distribution with a width of 0.3 and down shift of 1.8. Differential expression analysis was performed using Limma’s moderated two-sided t-test. The adjusted P values, using Benjamini–Hochberg’s procedure, were used for further analysis and visualization. Gene Ontology category enrichment analysis of the significantly dysregulated proteomic datasets was done using Shiny GO server as demonstrated in the Figure legends (43).

### Statistical analysis

Data are provided as arithmetic means ± SEM using GraphPad Prism, Version 8. Statistically significant differences between two groups were determined with a Students’ t-test, including Welch’s correction if indicated. Statistically analysis between several groups were determined using a two-way ANOVA, including Tukey correction. Significance was calculated as follows p > 0.05: n.s.; p < 0.05: *; p < 0.01: **; p < 0.001: ***; p < 0.0001: ****. *In vitro* assays were performed at least in three independent experiments.

## Results

### Phenotype expansion and survival beyond infancy with hypomorphic alleles

#### General features

Here, we report 22 novel patients from 20 families (**Table 1**). The female-to-male ratio was at 1.4 (females 13, males 9). The last reported age of the patients ranged from fetal demise at 28th week of gestation until 20 years. While 15 patients were reported as alive on last examination, seven patients were reported with an early demise, with the age at death ranging from *in utero* (28th week of gestation) to 7 years. Consanguinity was found in 73% (16/22). The ethnicities of the patients included Arabic (16/22), Afghan (1/22), European (2/22), Turkish (1/22), Indian (1/22), and Asian Indian (1/22).

**Table 1:** Patient IDs are indicated as P# for the newly described cohort, with the corresponding #-number referring to the same individual in the complete cohort of 74 patients for genotype-phenotype correlation study listed in **Supplementary Table 1** (P1-P22 and as #50–67 and #71–#74).

Patient	P5, #54	P19, #71	P6, #55	P7, #56	P8, #57	P9, #58	P10, #59	P11, #60	P12, #61	P13, #62	P14, #63	P15, #64	P17, #66	P18, #67	P20, #72	P21,#73	P1, #50	P16, #65	P4, #53	P3, #52	P2, #51	P22, #74
Domain	D1 CBM1	D2 CBM1													D3 Ig-like			D4 CBM2	D5 CBM2		D8 (FNIII)	NA
cDNA	c.194G>C	c.653dup													c.756dup	c.756dup, c.2444C>G	c.808T>G	c.1122-1_1122insAAAG	c.1438-4A>G	c.1469C>G	c.2497 + 1G>A	Deletion of LIFR exons 17-20
Protein	p.Cys65Ser	p.Glu219GlyfsTer3													p.Lys253Ter	p.Lys253Ter, p.Thr815Arg	p.Cys270Gly	p.Ser374ArgfsTer9 p	p.?	p.Ser490Ter	p.?	NA
Zygosity	Homozygous	Homozygous													Homozygous	Compound heterozygous	Homozygous	Homozygous	Homozygous	Homozygous	Homozygous	Homozygous
gnomAD	N	N	N	N	N	N	N	N	N	N	N	N	N	N	N	N; 1/152134	N	N	1/151968	N	N	NA
Consanguinity	Y	Y	Y	Y	Y	Y	Y	Y	N	N	Y	Y	N	Y	Y	N	Y	Y	Y	N	N	Y
Sex	F	F	F	M	F	M	M	M	M	F	F	F	F	F	F	M	F	M	F	M	M	F
Ethnicity	Arabic	Arabic	Arabic	Arabic	Arabic	Arabic	Arabic	Arabic	Arabic	Arabic	Arabic	Arabic	Arabic	Arabic	Romanian	Romanian	Afghan	Arabic	Turkish	Indian	Asian Indian	Dari/Persia
Age of last follow-up	9m	7y	8y	7y	3m	4y	4y	2m	20m	20m	20y	18y	3y 6m	3m	4y 8m	7y	6y 8m	79d	15y	*In utero* 28 gest. w	19d	5y 10m
Alive or dead	Alive	Alive	Alive	Died 7y	Died 3m	Alive	Alive	Died 2m	Alive	Alive	Alive	Alive	Alive	Alive	Died 4y 8m	Alive	Died 6y8m	Died 79d	Alive	Fetal death	Alive	Alive
Family	N	SWS	SWS	SWS	SWS	N	SWS	SWS	SWS	SWS	SWS	SWS	SWS	SWS	SWS	SWS	SWS	SWS	SWS	SWS	SWS	SWS
Perinatal features																						
Oligohydramnios	N	N	ND	N	ND	N	N	Y	N	N	ND	ND	Y	N	Y	ND	N	N	N	N	N	ND
Ophthalmological	N	Y	Y	Y	ND	Y	Y	ND	Y	Y	Y	Y	Y	N	Y	N	Y	N	ND	N	N	ND
Craniofacial	Y	Y	Y	Y	ND	Y	Y	ND	Y	Y	Y	Y	Y	N	Y	N	Y	Y	Y	Y	Y	Y
Muscular hypotonia	Y	Y	ND	N	ND	Y	ND	Y	Y	Y	ND	ND	N	N	Y	Y	Y	Y	Y	NA	Y	Y
Hyporeflexia	N	Y	Y	Y	ND	Y	Y	ND	ND	ND	Y	Y	Y	ND	Y	Y	N	N	N	NA	ND	N
Disease courses																						
NDD	Motor	Motor, speech	Motor, speech	Motor, speech, cognitive	N/A	Motor, cognitive	Motor, speech, cognitive	N/A	Motor, speech	Motor, speech	Speech	Speech	N	ND	Motor, speech, cognitive	Motor, speech	Motor, speech, cognitive	Motor	Motor, speech	NA	ND	Motor, speech, cognitive
Neurodegeneration	N	N	N	Y	N	Y	N	N	N	N	N	N	N	N	Y	N	Y	N	N	NA	ND	N
Dysautonomia	N	Y	N	Y	N	Y	N	N	Y	Y	N	N	Y	Y	Y	N	Y	Y	Y	NA	ND	Y
Feeding difficulties	Y	Y	Y	Y	N	Y	Y	Y	Y	Y	Y	Y	Y	Y	Y	Y	Y	Y	Y	NA	Y	Y
Immunodeficiency	Y	Y	N	Y	Y	Y	N	N	N	Y	Y	Y	Y	Y	Y	Y	Y	Y	N	NA	Y	Y
Allergies	N	N	N	Y	Y	N	Y	N	Y	Y	Y	Y	ND	ND	Y	N	N	N	N	NA	N	N
Skin	N	Y	N	N	N	N	N	N	N	N	Y	Y	Y	ND	Y	Y	N	N	N	ND	N	Y
Pulmonary	Y	Y	N	Y	Y	Y	N	Y	Y	Y	Y	Y	Y	Y	Y	N	Y	Y	N	ND	Y	Y
Cardiac	Y	N	Y	Y	N	N	Y	N	N	Y	Y	N	ND	ND	N	N	Y	Y	N	ND	Y	N
Short stature	Y	Y	Y	Y	Y	Y	Y	Y	Y	Y	Y	Y	Y	Y	Y	Y	Y	N	Y	ND	ND	Y
Skeletal	Y	Y	Y	Y	Y	Y	Y	Y	Y	Y	Y	Y	Y	Y	Y	Y	Y	N	N	Y	Y	Y
Kidney	N	Y	N	Y	N	Y	N	ND	ND	N	Y	ND	ND	ND	N	N	Y	N	N	ND	ND	N
Gastrointestinal	Y	Y	N	N	N	N	N	N	N	N	N	N	ND	ND	N	N	N	ND	N	Y	N	Y
Endocrinological	N	Y	ND	N	ND	N	ND	ND	N	N	N	N	ND	ND	N	Y	N	ND	N	ND	N	N

Cohort of 22 novel patients with *LIFR*-related disorders with variants on gene and protein, consanguinity, sex, ethnicity, age at last follow-up examination, status alive or dead, family history of SWS, oligohydramnios, ophthalmological features, craniofacial dysmorphia, muscular hypotonia, hyporeflexia, neurodevelopmental disorders (NDD), neurodegenerative features (spasticity, epilepsy), dysautonomia, feeding difficulties, immunodeficiency, allergies, skin features, pulmonary features, cardiac features, short stature, skeletal features, kidney features, gastrointestinal features, and endocrinological features. 1. Homozygous *LIFR* variants according to NM_001127671.2, ENST00000453190.7. Protein LIFR mutations according to NP_001121143.1, ENSP00000398368.2. In P3, the parents were first cousins, each heterozygous for c.1469C>G; p.Ser490Ter, fetal testing after *in utero* death was not performed according to parental wishes, (and the molecular genetic reported this *LIFR* variant as pathogenic as previously reported cases had been annotated as pathogenic in the ClinVar database (variation ID: 1070966)). Population database counts according to gnomAD v3.1.2. M, male; F, female, y, years; m, months; d, days; Y, yes; N, no; ND, not defined; NA, not available (due to fetal death).

#### Immunological disorders

Primary immunodeficiency was identified in 73% of cases (16/22) and presented with recurrent bacterial, fungal and viral lower respiratory tract infections (12/22, 55%), eye (7/22, 32%) and skin infections (5/22, 23%). Dental decay was noted in three patients. Atopic diseases were present in 36% of cases (8/22), mostly presenting with allergic asthma. Skin abnormalities were found in 27% of cases (6/22) and included xerosis cutis, seborrheic dermatitis, and delayed wound healing.

Patient P1 carrying a homozygous variant in p.Cys270Gly had a COVID-19 infection that led to acute cardiorespiratory failure within 12 hours of symptom onset, failed resuscitation in the emergency room, and death at six years and eight months of age. Patient P13 with a homozygous variant in p.Glu219GlyfsTer3 presented with COVID19-related multisystem inflammatory syndrome in children (MIS–C) at the age of 20 months that led to acute cardiorespiratory failure with resuscitation, acute kidney injury, and coagulopathy.

#### Skeletal abnormalities

Four cases died *in utero* or during infancy. Skeletal features were observed in all cases (22/22) and included bowing of the long limbs in 16 cases, camptodactyly in 13 cases, scoliosis in eight cases, osteopenia in four cases, hip dysplasia in four cases, shortened upper and/or lower limbs in four cases (**Figures 1D, H, I–O**). Short stature was found in 86% of cases (19/22). Moreover, those deformities often progressed throughout life and resulted in several orthopedic surgeries and/or immobility.

**Figure 1:**
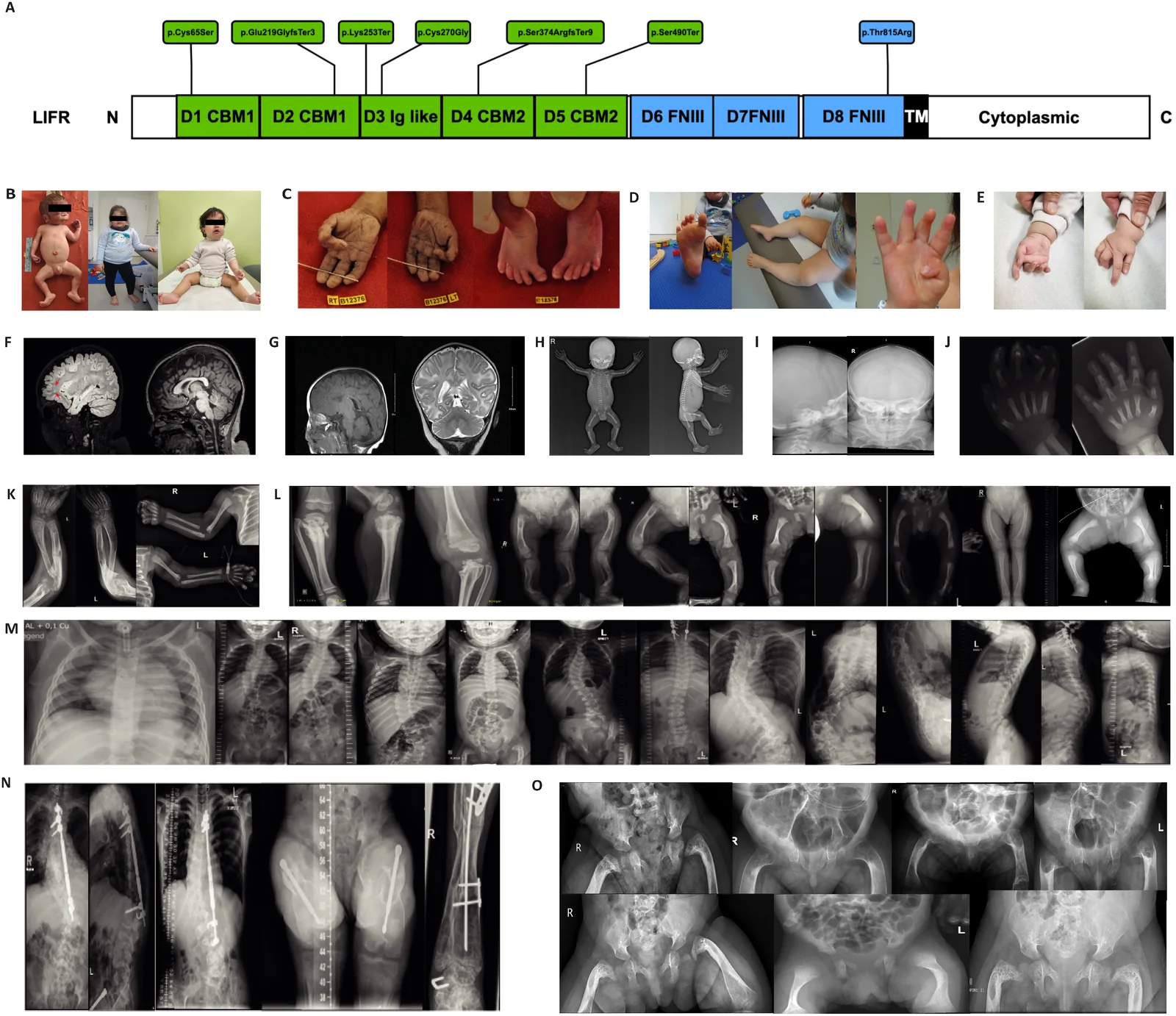
Phenotypic and imaging spectrum of 22 novel patients with *LIFR*-related Stüve-Wiedemann syndrome. **(A)** Mutations on LIFR protein structure. **(B)** Three patients with *LIFR*-related disorders illustrate the spectrum in disease severity, left P3 (#52) *in utero* onset with fetal akinesia and death, middle P1 (#50) patient at six years and onset at infancy with skeletal dysplasia, immunodeficiency, and NDD, and right P5 (#54) patient at 9 months with typical SWS features. **(C)** Contractures in hands and feet with *in utero* onset in P3. **(D)** Skeletal dysplasia with bowing of long femoral bones, contractures in hands and feet in P1. **(E)** Camptodacyly of right hand in P5. **(F)** MRI revealed FLAIR hyperintensities marking brain inflammation (left) and brain atrophy with mild corpus callosum dysgenesis (right) in P1. **(G)** MRI revealed few foci of hemosiderin deposition within the choroid plexuses in P5. **(H)** X-rays from antenatally deceased P3 indicating skeletal dysplasia with bowing of long bones and contractures. **(I)** X-ray of skull showing dolicocephaly. **(J)** X-ray indicating left hand contractures. **(K)** Skeletal dysplasia with bowing of radial and ulnar bones. **(L)** Skeletal dysplasia with bowing of tibial and femoral bones. **(M)** X-rays indicating cardiomegaly and scoliosis. **(N)** Surgical correction of scoliosis and femoral bowing using elongating rods and material. Lower leg treated with a rod and two plates. **(O)** Skeletal dysplasia with developmental dysplasia of hips.

#### Neurological disorders

Neurodevelopmental disorders (NDD) were found in 73% of cases (16/22), most frequently with motor delay (15/22, 68%), speech delay (13/22, 59%), and intellectual disability (6/22, 27%). Of note, two patients only had speech delay but age-adequate motor and cognitive milestones. In two patients carrying the *LIFR* variants p.Cys270Gly and p. Lys253Ter, we observed corpus callosum dysgenesis, brain atrophy and multiple FLAIR-hyperintensities on brain magnetic resonance imaging (MRI) (**Figures 1F, G**). Neurodegenerative features were present (4/22, 18%) and included spastic paraplegia and epileptic encephalopathy. Dysautonomia was present in 55% of cases (12/22) and included tongue ulcerations (n=2), hyperthermia (n=6), hypothermia (n=1), and paradoxical sweating (n=3). One patient suffered from rhabdomyolysis during a hyperthermic event.

### Population genetics and molecular genetic spectrum in milder disease

The genotypes in our patient cohort (n=22) included four truncating variants, two splice site variants, and three missense variants (**Table 1**; **Figure 1A**). Only two of the above variants were present in the population database gnomAD v 3.1.2 (c.1438-4A>G with 1/151,968 in heterozygous state and c.2444C>G,p.Thr815Arg with 1/152,134 in heterozygous state; no homozygotes were recorded for any of these variants). The other variants were absent from population databases.

In first analyses, we examined a potential association of the predicted variant effect on protein (missense vs. truncation) with patient survival. Seven patients were reported as deceased. We observed homozygous truncating variants in six of these patients and a homozygous missense variant in one patient.

Upon analysis of the variant localization within specific protein domains, we observed that patients with variants affecting the C-terminal half of the extracellular region showed early demise, with age of death ranging from 28th week of gestation (p.Ser490Ter), to 19 days (c.2497 + 1G>A) and 79 days postnatally (p.Ser374ArgfsTer9), respectively. The patient with *in utero* death and fetal akinesia is shown in **Figure 1B** (left) and 1C. A 15-year-old patient with a variant in D5 CBM2 (c.1438-4A>G) had severe life-threatening events of paradoxical sweating requiring intensive care treatment. The patient with a variant in D4 CBM2 (p.Ser374ArgfsTer9) presented with an episode of severe rhabdomyolysis upon infection at 79 days of age. The **Supplementary Material** shows a genotype-phenotype correlation analysis with all published patients.

### N-terminal *LIFR* missense variants reduce and C-terminal variants abolish protein function

As we observed several cases where early death was associated with variants in the C-terminal half of the extracellular region, we examined whether the localization of missense variants had any effect on functional activity.

In Ba/F3-gp130 cellular proliferation assays, stimulation with LIF (0.00001–100 ng/ml) showed reduced proliferation for all LIFR mutants up to 0.01 ng/ml except for p.Arg88His when compared to Ba/F3 cells expressing the wild-type proteins (**Figure 2A**). With LIF stimulation above 0.01ng/ml, all missense variants in the extracellular regions showed markedly reduced proliferation, whereas variants in the cytoplasmic region did not proliferate at all. EC50 levels were elevated for LIFR p.Asn128Lys and LIFR p.Gly779Val (**Figure 2B**).

**Figure 2:**
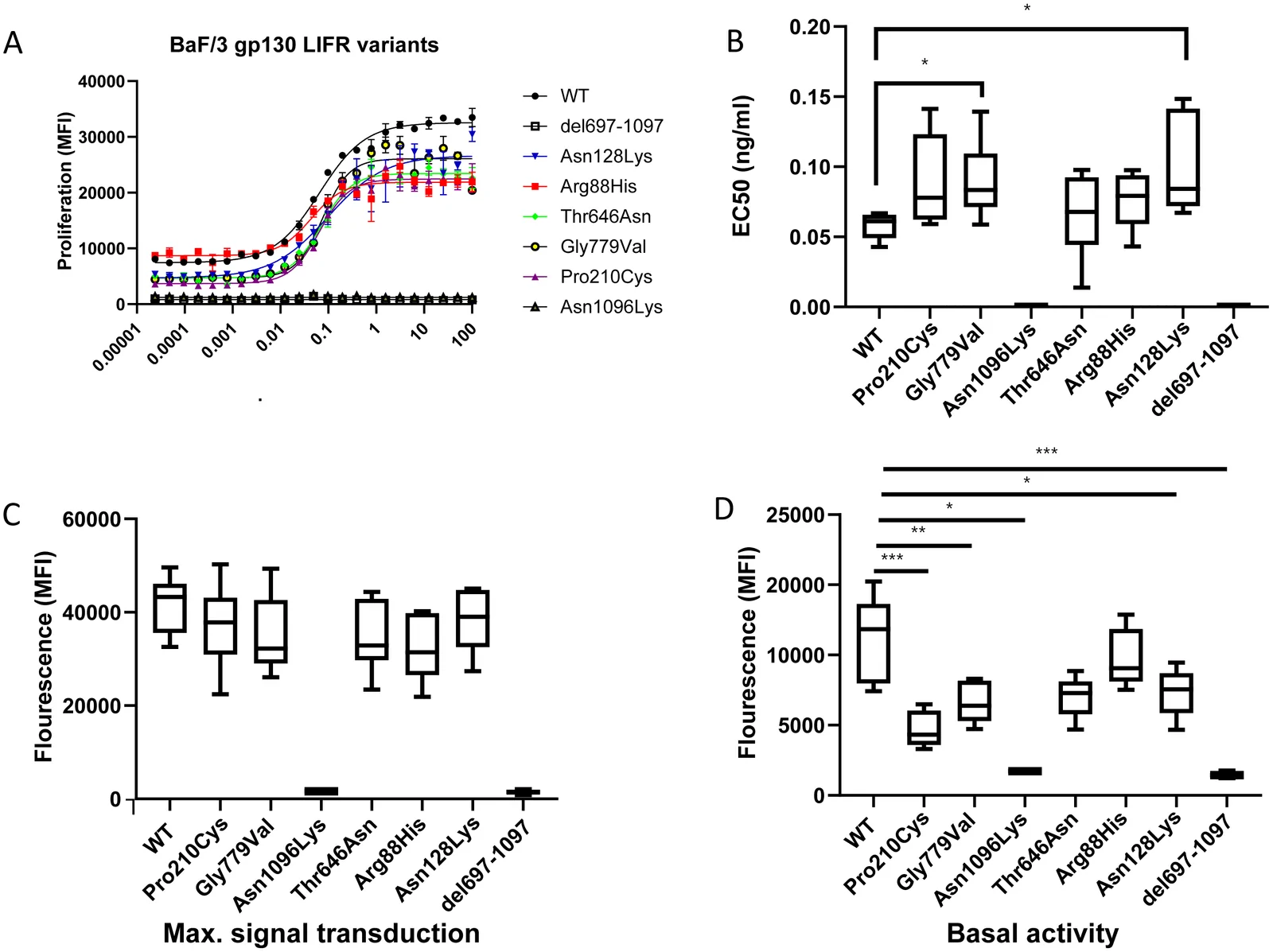
Allelism in LIFR reveals marked reduction in cellular proliferation and complete loss of function in cytoplasmic region variants. **(A)** Cellular proliferation assay indicating mean fluorescence intensity (MFI) after increasing amounts of LIF stimulation (0.00001–100 ng/ml) in Ba/F3 cells with wildtype gp130/IL6ST and LIFR (WT) and LIFR patient alleles (p.Tyr697Ter, p.Asn128Lys, p.Arg88His, p.Thr646Asn, p.Gly779Val, p.Pro210Cys, p.Asn1096Lys) indicate reduced proliferation associated with variants in the extracellular region and absent proliferation associated with variants in the cytoplasmic region (p.Tyr697Ter, p.Asn1096Lys). **(B)** EC50 measurements in proliferation assay indicate significantly higher concentrations of LIF for cellular proliferation in patient alleles p.Gly779Val and Asn128Lys, a non-significant but increased EC50 in the other extracellular region variants, and notably completely absent values in the cytoplasmic region variants, when compared to wildtype. **(C)** Maximum signal transduction as measured by MFI is reduced in extracellular region variants and notably absent in cytoplasmic region variants. **(D)** Basal activity is significantly reduced in most extracellular region variants and almost absent in cytoplasmic region variants. All experiments were done in in technical triplicates and analyzed in two-way-ANOVAs with Tukey correction. p < 0.05: *; p < 0.01: **; p < 0.001: ***; p < 0.0001: ****.

In Ba/F3-gp130 cells expressing *LIFR* variants with mutations in the extracellular region, maximal signal transduction was largely unaffected (**Figure 2C**). However, basal activity was significantly reduced with *LIFR* variants in the extracellular region p.Asn128Lys, p.Pro210Cys, p.Thr646Asn, and p.Gly779Val, but notably unremarkable with p.Arg88His in comparison to wild type (**Figure 2D**). This finding suggests that missense variants in the N-terminal part of the extracellular region may not have a severe effect on basal activity which correlates with the less severe phenotype in patients with N-terminal *LIFR* variants.

Finally, we examined one missense variant in the cytoplasmic region (p.Asn1096Lys) as well as a deletion of the cytoplasmic region (p.Tyr697Ter). These variants in the cytoplasmic region have notably shown both absent maximum signal transduction and basal activity, causing complete loss-of-function in LIFR. These findings correlate with severe phenotypes and early death in *LIFR* patients with variants in the C-terminal half of *LIFR* as well as the noted absence of biallelic variants in the cytoplasmic region which may not be viable in human (**Figures 2A–D**).

### *LIFR*:p.Cys270Gly missense variant leads to LOF with defective STAT and ERK signaling

Most research on human LIFR deficiency was previously focused on biallelic truncating variants, we examined the biallelic missense mutation p.Cys270Gly in a murine cell model. We picked this particular p.Cys270Gly variant in the D3 Ig-like domain as it was identified in a patient (P1) who survived infancy, gave bronchoalveolar cells during early childhood and later died at 6 years and 8 months of age during a COVID-19 infection. We were interested in this variant as it localized to a protein region which, if mutated, appeared to be associated with better overall life expectancy compared to variants in the D4-D6 domains but associated with a higher risk of infection-associated deterioration and death.

Proliferation of the murine pre-B cell line Ba/F3 is IL-3 dependent (41) The variant p.Cys270 is located in D3 of the LIFR, forming a disulfide bond to p.Cys163 in D2. To investigate the impact of the mutation on cell proliferation we performed a dose-dependent proliferation assay. Ba/F3 cells expressing the wildtype LIFR:gp130 receptor complex proliferate LIF-dose-dependently with a EC50 of 51 pg/ml, whereas Ba/F3 cells expressing the p.Cys270Gly variant proliferate with an EC50 of 639 pg/ml (**Figures 3A, B**). To analyze the STAT3 phosphorylation, Ba/F3 cells expressing gp130, gp130-LIFR or gp130-LIFR p.Cys270Gly were stimulated with HIL-11 or increasing amounts of LIF. Western blotting showed that the same amounts of LIF (10 ng/ml) led to increased STAT3 phosphorylation, if the wildtype LIFR receptor was expressed, compared to the p.Cys270Gly protein (**Figures 3C, D**).

**Figure 3:**
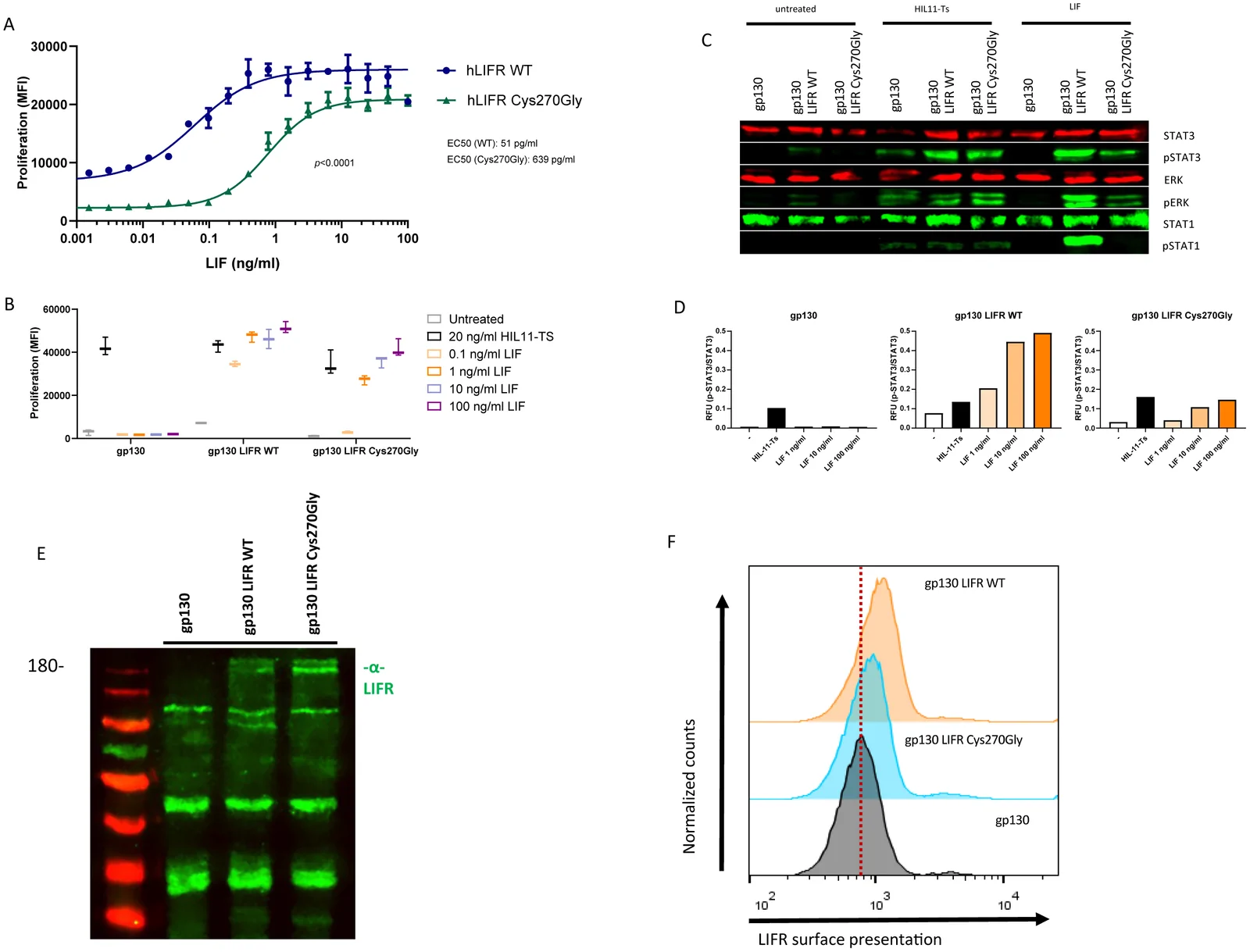
Cellular assays on the missense variant p.Cys270Gly from P1. **(A)** Proliferation assay with Ba/F3-gp130-LIFR (blue) and Ba/F3-gp130-LIFR Cys270Gly (green) cells from four biological replicates and three technical replicates in the presence of increasing concentrations of LIF (0.001–100 ng/ml) indicates that significantly higher concentrations of LIF is required for proliferation of mutant cells than wildtype cells. One representative experiment is shown, p<0.0001 in two-way ANOVA with Tukey correction. **(B)** Cellular proliferation assay with Ba/F3-gp130, Ba/F3-gp130-LIFR and Ba/F3-gp130-LIFR Cys270Gly cells with three technical replicates, treated with LIF (0.1, 1, 10 and 100 ng/ml), HIL11-Ts (20 ng/ml) and untreated, show that stimulation is dependent on presence of LIFR in gp130 cells and reduced in mutant LIFR indicating loss-of-function. **(C)** Ba/F3-gp130, Ba/F3-gp130-LIFR and Ba/F3-gp130-LIFR Cys270Gly cells in one representative experiment without cytokine (-), HIL-11-Ts (50 ng/ml) and LIF (10 ng/ml) after 15 min stimulation. HIL-11-Ts (Hyper-Interleukin-11 trans-signaling construct) binds to and activates signaling via the standard IL6ST (gp130) co-receptor, but it does not utilize or signal through LIFR in its native configuration. Instead, HIL-11-Ts acts as a potent agonist for conventional IL-11/gp130 receptor assemblies, driving robust downstream JAK/STAT (such as STAT3) activation and cell proliferation strictly through IL6ST (gp130) and IL-11R components, unassisted by LIFR. While HIL-11-Ts induced detectable gp130-dependent signaling, LIF stimulation resulted in a broader activation of downstream signaling pathways including STAT1. The p.Cys270Gly variant does not lead to STAT1 phosphorylation and only reduced STAT3 phosphorylation. ERK phosphorylation is notably reduced on LIF stimulation but not on Hyper-IL-11 stimulation. Equal amounts of proteins (50 µg/lane) were analyzed via specific antibodies detecting phospho-STAT3, STAT3, phosphor-ERK, ERK, phosphor-STAT1 and STAT1. **(D)** STAT3 phosphorylation upon LIF stimulation is dependent on presence of LIFR in gp130 cells and reduced in mutant LIFR indicating loss-of-function. Quantified relative fluorescence units of phospho-STAT3 divided by total STAT3 signal derived from the stimulation assay followed western blot. STAT3 activation of Ba/F3-gp130 cells (left), Ba/F3-gp130-LIFR (center) and Ba/F3-gp130-LIFR Cys270Gly (right) cells without cytokine (-), HIL-11-Ts (50 ng/ml) and with increasing amounts of LIF (1, 10, 100 ng/ml) after 15 min stimulation. Equal amounts of proteins (50 µg/lane) were analyzed via specific antibodies detecting phospho-STAT3 and STAT3. **(E)** Mutant protein is expressed in similar amounts when compared to wildtype LIFR protein (n=1 experiment). Ba/F3 gp130 and stable transduced Ba/F3 lysates were used for the detection of LIFR via specific antibodies under reducing and non-reducing conditions. Equal amounts of proteins (50 µg/lane). **(F)** The p.Cys270Gly variant is less presented on the cell surface as indicated with one flow cytometry experiment via specific antibodies.

With respect to the STAT3 phosphorylation in Ba/F3-gp130 cells expressing the LIFR variants, phosphorylation of STAT1 and STAT3, as well as downstream MAPK/ERK pathway activation were investigated after stimulation with either HIL-11 or LIF. Ba/F3-gp130 cells expressing wildtype LIFR induce STAT3 phosphorylation more efficiently than cells expressing the LIFR p.Cys270Gly variant. Concomitantly with STAT3 phosphorylation also ERK phosphorylation is less efficiently transduced by LIFR p.Cys270Gly. Interestingly, intracellular STAT1 phosphorylation could not be induced by the p.Cys270Gly variant of LIFR, using a high concentration of LIF (**Figure 3C**).

To investigate total expression, stably transduced Ba/F3-gp130-LIFR and Ba/F3-gp130-LIFR p.Cys270Gly cells were lysed and LIFR was detected by Western blotting, showing the same amount of LIFR p.Cys270Gly compared to the wildtype receptor (**Figure 3E**). To analyze the density of expressed LIFR on the cell surface, flow cytometry was performed, showing a lower surface presentation of the LIFR p.Cys270Gly variant compared to the wild type (**Figure 3F**). The hypomorphic p.Cys270Gly variant allows only weak STAT3 phosphorylation but shows a lack of STAT1 phosphorylation upon LIF stimulation.

### Patient p.Cys270Gly bronchoalveolar cells show endolysosomal trafficking dysregulation

To explore impact of a *LIFR* missense variant in patient-derived cells, we conducted LC-MS/MS proteomics on primary patient cells obtained through bronchoalveolar lavage fluid from the patient with the p.Cys270Gly variant and a healthy age- and sex-matched control without Stüve-Wiedemann syndrome (**Figure 4A**); the patient and control did not have any infection at the point the sample was taken. Of note, TMEM173/STING was upregulated in patient bronchoalveolar cells, already suggesting STING upregulation at a time without clinical infection likely due to chronic hyperinflammation.

**Figure 4:**
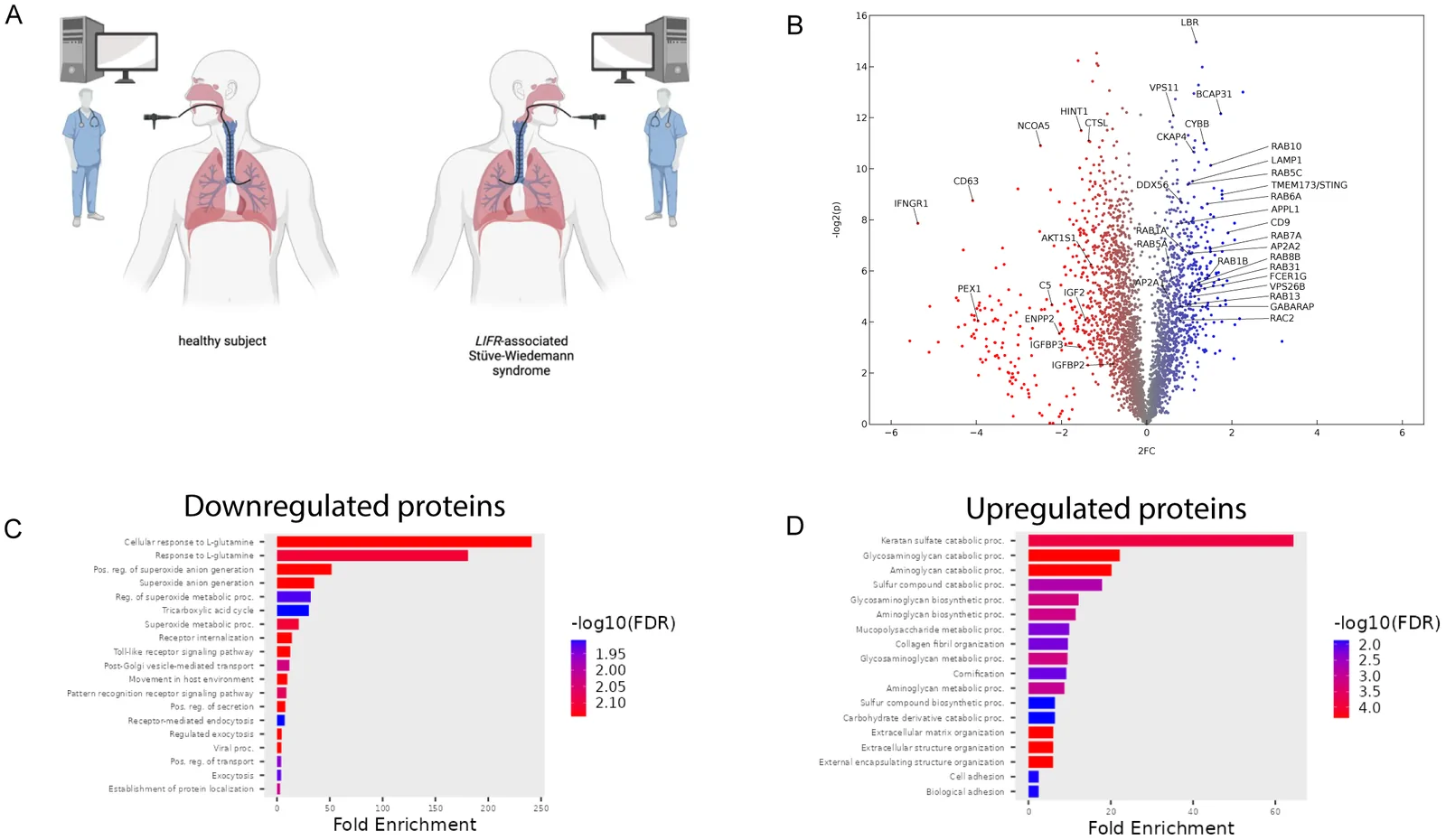
Proteomics in bronchoalveolar cells of one patient with a *LIFR*-related disorder and a LOF p.Cys270Gly variant shows dysregulation in vesicular trafficking when compared to one healthy age-matched control. **(A)** Overview of patient and healthy control sampling from bronchoalveolar lavage fluid. **(B)** Volcano plot indicating upregulation of vesicular trafficking proteins (RAB1, RAB5, RAB6, RAB7, RAB10, BCAP31, LAMP1) and TMEM173/STING. We also observed downregulation of cathepsin L (CTSL), the neuropathy-associated protein HINT1, immunological proteins (IFNGR1, CD63, NCOA5) and proteins involved in body growth (IGFBP3, IGFBP1, IGF2). Color coding from red (downregulated) to grey to blue (upregulated). Only proteins above -log2(p) above 2.0 were annotated, while 2FC (2-fold change) values were below -0.5 or above 0.5. **(C)** Downregulated pathways for Gene Ontology biological process terms indicate response to amino acids and toll-like receptor pathway. Color coding for -log10(FDR) from blue to red with greater significance. **(D)** Upregulated pathways for Gene Ontology biological process terms indicate catabolic processes for keratan sulfate, glycosaminoglycans, and aminoglycans. Color coding for -log10(FDR) from blue to red with greater significance.

We also observed dysregulation of downstream endocytosis trafficking, including significant upregulations in the AP2 subunits AP2A1 and AP2A2, the endosome-associated adaptor protein APPL1 as a Rab5 effector protein, and the Rab5 proteins RAB5A and RAB5C that are involved in maturation of early endosomes to lysosomes. Additional findings in upregulation of endosome-associated Rab proteins include the proteins associated with trafficking from ER to Golgi (RAB1A, RAB1B, RAB6A), trafficking from Golgi to early endosomes (RAB31, AP3D1, GABARAP), trafficking of lipid droplets (RAB18), and trafficking from plasma membrane to endosomes (RAB10, RAB13). A notable finding regarding a stall in late endosomes/multivesicular bodies is indicated by upregulation of CD9 and downregulation of CD63.

Other upregulated Rab proteins indicated a stall in later stages of endolysosomal trafficking, including RAB35 involved in selective autophagic clearance of bacteria (xenophagy) and mitochondria (mitophagy) as well as RAB7A. Notably, VPS11 and LAMP1 were upregulated indicating a stall in autophagolysosomal fusion, while cathepsin L (CTSL), a direct transcriptional target of STAT3, was significantly downregulated, also indicating a stall in autophagolysosomal flux.

We also noted downregulation in immunological proteins including IFNGR1, and NCOA5, as well as in proteins involved in growth including IGFBP3, IGFBP1, and IGF2 (**Figure 4B**).

Gene Ontology molecular function terms indicated downregulation in the pathways for amino acid responses and the toll-like receptor pathway, and upregulation in pathways for catabolic processes for keratan sulfate, glycosaminoglycans, and aminoglycans (**Figures 4C, D**).

## Discussion

We present the largest cohort of patients with *LIFR*-related Stüve-Wiedemann syndrome reported to date (n=22), prompting several milder presentations with survival beyond infancy and *LIFR* missense variants. We leveraged a murine cellular model to assess effects of missense variants in LIFR on cellular proliferation upon stimulation with LIF. While some of the variants were found in patients with mild or moderate phenotypes (p.Thr646Asn, p.Gly779Val, p.Asn1096Lys), we also examined missense variants either in amino acid residues that were previously published with truncating variants, including p.Asn128Lys for p.Asn128LysfsTer2, or located next to published variants, including p.Pro210Cys for the founder variant p.Glu219GlyfsTer3. While the N-terminal variant p.Arg88His did not reveal a significant change in comparison to wildtype, all other variants did indeed confer deleterious effects on cellular proliferation and basal receptor activity of LIFR. To investigate the effects on the cytoplasmic region, we examined a *LIFR* variant with a large truncation of the cytoplasmic region (p.Tyr697Ter) which displayed an absence of proliferation and basal receptor activity of LIFR. LIF signaling requires a functional receptor complex consisting of LIFR and IL6ST (gp130), with both receptor subunits contributing to downstream signal transduction (44). The absence of a proliferative response observed for the *LIFR* variant affecting the intracellular domain suggests that the presence of IL6ST is not sufficient to fully compensate for impaired intracellular LIFR signaling. This may indicate that the intracellular domains of both receptor proteins contribute to effective signal transduction, although the precise interplay and relative contributions of LIFR and IL6ST remain to be elucidated (45). When including published reports with *LIFR*-associated SWS (74 patients from 64 families, **Supplementary Material**), we observed that cytoplasmic region variants in *LIFR* were only found in heterozygosity, likely due to its essentiality for cellular viability upon infection (46), and further research may be required to determine whether any homozygous variant in that region may be viable in human.

In addition, our data suggest a tentative association between genotype and phenotype with regards to survival. The seven deceased patients carried homozygous truncating variants (n=6) and a homozygous missense variant (n=1) with variants located in domains D2, D3 or D4. However, the limited allelic diversity in this cohort, which is dominated by a recurrent founder variant and may be related to the shared ethnic background of many individuals, and the variability in survival among individuals carrying the same variant indicates that factors beyond variant location contribute to clinical outcome. These may include genetic background, consanguinity, family history of SWS, access to specialized medical care, and regional differences in healthcare. Although our functional data suggest more severe consequences of *LIFR* variants in the cytoplasmic region, domain location alone should not be considered a reliable predictor of survival. Of note, 73% of individuals in our cohort were born to consanguineous parents, and the cohort presents a strong predominance of individuals of Arabic ethnicity that may present a bias. Further studies in more ethnically and geographically diverse cohorts will be required to validate these observations.

We focused on the homozygous missense variant p.Cys270Gly in the D3 domain, found in a patient with several signs of classic Stüve-Wiedemann syndrome who survived infancy but succumbed to COVID-19-MIS-C at the age of six years and eight months. To confirm the pathogenicity of this variant, our work in Ba/F3 cells with this mutation confirmed (i) reduced cellular proliferation and phosphorylation of STAT3 and ERK after treatment with LIF, (ii) absent phosphorylation of STAT1 after treatment with LIF, and (iii) reduced LIFR cell surface expression by flow cytometry indicating a lower surface presentation of the LIFR p.Cys270Gly variant compared to wild type. As total LIFR expression in cells was similar to wildtype, we noted that this variant leads to altered LIFR protein localization from the cellular surface to the cytoplasm by endocytosis, ultimately prompting lysosomal degradation for recycling. Mechanistically, endocytosis and lysosomal degradation of LIFR are mediated by the adaptor protein AP2 and the kinase ERK (47). The proinflammatory cytokine tumor necrosis factor alpha (TNFa) downregulates LIFR expression and injection of lysosomal inhibitors including chloroquine rescue LIFR expression (48). We present the first study that shows reduced ERK activity in human LIFR deficiency. While previous studies have shown reduced STAT3 phosphorylation with *LIFR*-SWS (2, 20, 49), only studies in mouse and cellular models have previously examined the effect on ERK (50, 51). LIFR loss-of-function in SWS may provide a distinct pathomechanism including dysregulated endolysosomal trafficking that is particularly relevant in the survival of post-mitotic tissue where cellular survival hinges upon degradation of dysfunctional cargo for protein quality control (52). We delineated phenotypes with core manifestations in the nervous system, the skeleton, and immune cells, which are marked by a rather high degree of post-mitotic cells for which protein and organellar quality control mechanisms are essential for viability. Of note, there is a distinct phenotypic overlap in patients with Stüve-Wiedemann syndrome and lysosomal or autophagy disorders, particularly with neurodevelopmental disorders, neuroinflammation, and short stature (53). Recent reports on defects in autophagolysosomal tethering with *EPG5*-related Vici syndrome with mitochondrial myopathy and neurodegenerative disease courses show an upregulation in STING in neuronal cells (54). Notably, we also found upregulation in TMEM173/STING in LIFR:p.Cys270Gly bronchoalveolar cells without a presence of an infection, supporting the notion of a chronic hyperinflammation in bronchoalveolar cells.

Loss of LIFR was previously reported to activate NF-κB signaling (55), eventually prompting STING activation. Our clinical data on milder *LIFR*-disorders (**Table 1**) show survival beyond infancy but myopathic and neurodegenerative disorders later in childhood, further aided by two reports of mitochondrial myopathy in SWS patients (35) and by neurodegeneration in histopathological analyses of *Lifr* knockout mice (56), while previous reports on truncating variants showed demise at infancy (15, 20, 32).

To gain additional insight into dysregulated endolysosomal trafficking, we chose the p.Cys270Gly variant for our proteomics analyses in patient bronchoalveolar cells. First analyses on key proteins involved in endolysosomal trafficking showed upregulations of the AP2 subunits AP2A1 and AP2A2 (**Figure 4**) indicative of increased endocytosis trafficking in patient bronchoalveolar cells, in line with the previous observation of AP2-mediated endosomal trafficking of LIFR (47). Another notable finding in this pathway was upregulation of the endosome-associated adaptor protein APPL1, a Rab5 effector previously identified in endosomal trafficking of LIFR (12). In line with this, we observed an upregulation in endosomal proteins (RAB5A/C) involved in maturation of early endosomes to lysosomes. This proteomic signature is further indicated by the upregulation of proteins associated with trafficking from ER to Golgi (RAB1A/B-RAB6A), trafficking from Golgi to early endosomes (RAB31-AP3D1-GABARAP), trafficking of lipid droplets (RAB18), and trafficking from plasma membrane to endosomes (RAB10-RAB13). This proteomic signature of endosomal dysregulation demonstrates an attempt to internalize dysfunctional LIFR for recycling. However, it shall be noted that this exploratory finding in the proteomic analysis of bronchoalveolar lavage (BAL) cells was performed in samples obtained from one patient and one control subject, and further proteomic analyses in larger cohorts are required in follow-up investigations.

Whereas, the notion of dysregulated endosomal trafficking response further extends to RAB35, involved in xenophagy and mitophagy (57), as well as RAB7A, VPS11 and LAMP1, involved in autophagolysosomal fusion (58), and notably, downregulation of cathepsin L. At physiological conditions, activated STAT3 upregulates cathepsin L, which enhances lysosomal membrane permeabilization for adequate autophagolysosomal flux (59). In a murine retinal degeneration model, inhibition of STAT3 signaling with niclosamide led to stalled autophagolysosomes (60). Notably, STAT3 phosphorylation leads to BNIP3 activation, a potent mitophagy regulator (61), raising the discussion whether, in turn, defective mitophagy may contribute to further waste accumulation. Upon investigating late endosomal proteins, we observed downregulation of CD63 and upregulation of its negative regulator CD9, both involved in endosomal fusion with the plasma membrane to ultimately release vesicles into extracellular space (62). STAT3 failed to localize to late endosomes in STAT3-mutant mice (63). In line with this, we have shown attenuated STAT3 activation upon LIF stimulation in p.Cys270Gly BaF/3 cells. Downregulation of exocytosis in light of reduced STAT3 activation may be particularly important for neuronal health in patients, in whom neurodegenerative manifestations include epileptic encephalopathy and spastic paraplegia (**Table 1**), similar to *TBCK*-related neurodevelopmental disorders where short stature with upregulations of RAB5 (64) indicate a common pathway to compensate for accumulation of intracellular waste.

Moreover, short stature remains a notable phenotype in the context of reduced STAT3 activation, as STAT3 deficiency (Job’s syndrome) is associated with similar skeletal anomalies as SWS, but usually STAT3 gain-of-function mutations are observed with short stature. Notable findings involved in growth-related proteins from our proteomics assays included downregulations in IGFBP3, IGFBP1, and IGF2. GO molecular function term analyses indicated upregulation of catabolic processes for keratan sulfate, glycosaminoglycans, and aminoglycans which are primarily involved in cartilage and the extracellular bone matrix. In addition, a mouse model of STAT3 deficiency shows bowing of long bones and short stature (65), perhaps analogous to the human SWS phenotype with reduced STAT3 phosphorylation in the above experiments for the p.Cys270Gly variant. The early spontaneous fractures and the hip cartilage defects in these STAT3-deficient mice may also be due to an increased catabolism of extracellular bone-matrix proteins. We discuss that short stature with bowing of long bones in SWS may be distinct from the short stature in STAT3 GOF.

Furthermore, the observed MIS-C in our patient with a biallelic p.Cys270Gly missense variant may serve as an important note for other patients with SWS. In a published report on COVID19-related patient plasma samples, LIFR expression correlated with significantly less hospitalization and lower progression to severe acute illness (66). IL6-receptor (IL-6R) antagonists such as tocilizumab have been used in the acute management of COVID19-related infection with variable efficacy, possibly accounted for by variants in the paralog gene *IL6ST*/gp130 (67). However, it is unclear whether tocilizumab would be beneficial in the setting of MIS-C with LIFR deficiency. As part of the life-threatening *LIFR*-related disorder, our clinical data indicate multiple bouts of infections including pneumonia (2), while intravenous immunoglobulin (IVIG) infusions were reported as beneficial with hypogammaglobulinemia in SWS type 2 individuals with variants in the paralog gene *IL6ST* (68, 69), and subcutaneous administration (SCIG) for *IL6ST*-related urticaria (70). While steroid treatment may also dampen hyperinflammatory processes with only anecdotal support, the monoclonal IgG4 antibody dupilumab was reported for *IL6ST*-related eczema (70). We also observed an upregulation in FCER1G, part of the high-affinity IgE receptor. Only *IL6ST*-related disorders were previously associated with elevated IgE levels (3). Triggering IgE receptors with anti-IgE-antibody led to a rapid release of LIF in human lung tissues (71). While it is conceivable that IgE elevation may be relevant in LIFR deficiency, there is no sufficient clinical data to correlate the human disease course with IgE elevation at this time.

Hyperthermia was presented mostly at infancy and early childhood in *LIFR*-related disease, and the life-threatening episodes were occasionally associated with an early death. We propose that patients should be monitored regularly by immunological services even after survival beyond infancy, in particular during intercurrent viral infections to prevent life-threatening events. In line with malignant hyperthermia in *RYR1*-associated neuromuscular disorders (72–74), the association of SWS with mitochondrial myopathy shall prompt that depolarizing medications are to be excluded in the care of patients with SWS (75), further highlighted by a report with rhabdomyolysis after anesthesia (76).

**Figure 5** illustrates the expanding phenotype of *LIFR*-disorders showing the overlapping and distinctive features towards *IL6ST*-disorders. Taken together, our studies in a murine cellular model and LC-MS proteomic analyses of human samples gave detailed insight into dysregulated pathways of endolysosomal trafficking, immune response and growth-associated proteins. The expanded phenotypes in our novel 22 patients will aid in genetic counselling and surveillance of patients and families.

**Figure 5:**
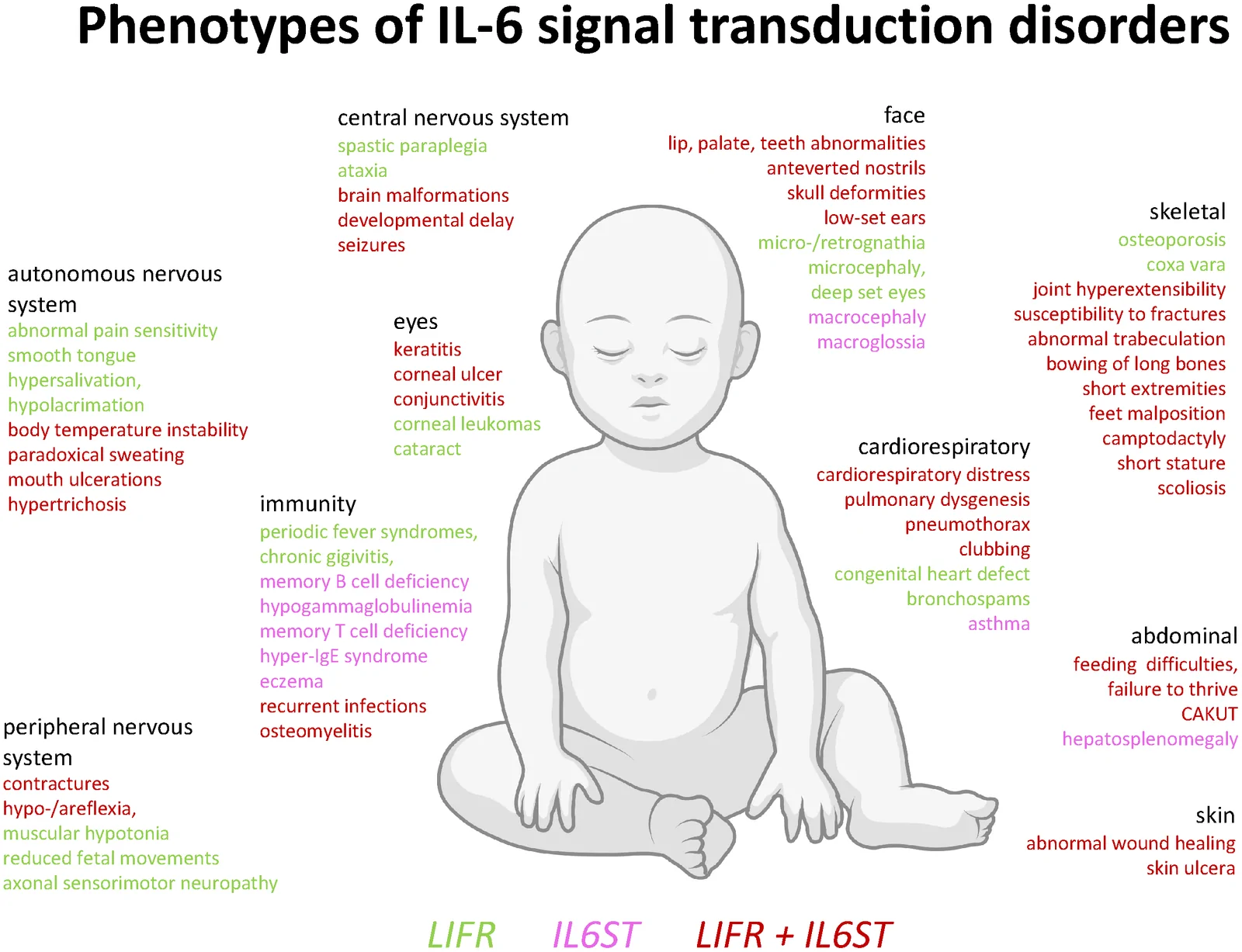
Overview of phenotypes in IL-6 signaling disorders. LIFR-related phenotypes in green, *IL6ST*-related in pink, and overlapping phenotypes in both *LIFR*- and *IL6ST*-related disorders in red. The phenotypic data were derived from a combination of our newly recruited *LIFR* cohort and previously published patients with LIFR and IL6ST deficiencies. The summarized phenotypic data comprise a total of 104 individuals, including 74 individuals with LIFR deficiency (22 newly recruited individuals and 52 previously published individuals) and 30 individuals with IL6ST deficiency (1 newly recruited individual and 29 previously published individuals). Phenotypic frequencies for the LIFR cohort are provided in **Supplementary Material**.

## Data Availability

The original contributions presented in the study are included in the article/**Supplementary Material**; further inquiries can be directed to the corresponding author/s. The raw proliferation data supporting the conclusions of this article are available from the authors upon reasonable request. Restrictions apply to the proteomics datasets: the datasets are not readily available because they were generated from patient samples and cannot be publicly shared due to privacy and re-identification concerns.
