# Supplementary material for: Hypomorphic biallelic LIFR variants define a multisystem disorder with survival beyond infancy and hyperinflammatory vulnerability
Source: Front Immunol. 2026 Oct 19;17:1927542. doi: 10.3389/fimmu.2026.1927542 (PMC13724599; doi:10.3389/fimmu.2026.1927542)
Supplement: Supplementary file 1 [file Table1.docx]

**Supplementary material**

**Analyses from published patients and our novel cohort**

These analyses were conducted in n=22 novel patients from this paper and previously published patients, as indicated in the methods section.

**Perinatal features.** The most frequent perinatal feature were limb contractures in 86% of cases (19/22) and generalized muscular hypotonia in 59% of cases (13/22) (Figure 1). Craniofacial dysmorphia was observed in 82% of cases (18/22) and included widened cranial sutures with asymmetric bilateral lambdoid Wormian bones, micrognathia, smooth philtrum, high-arched palate, short palpebral fissures, depressed and wide nasal bridge with a short nose, frontal bossing, square face, midface hypoplasia, pursed lips, hypertelorism, low set ears, and exophoria. Ophthalmological features were observed in 41% of cases (9/22) with corneal defects in eight cases ranging from corneal opacity to corneal scars and entropion, keratoconjunctivitis sicca (n=6), dry eyes (n=2), esotropia (n=2), exotropia (n=1), cataracts (n=2), amblyopia (n=1), and retinopathy and idiopathic blindness in one case each, respectively. Lack of reflexes in the peripheral and central nervous system was observed in 44% of cases at birth (10/22) with absent corneal reflexes in nine cases and absent patellar reflexes in six cases. Oligohydramnios was observed in 14% of cases (3/22).

**Neurological disorders** were observed in patients with variants in extracellular domains but without a clear association to a specific region. Motor NDD was frequently observed with variants in D3 (3/3, 100%), D2 (7/13, 54%), and speech NDD was most frequently observed with variants in D3 (3/3, 100%) and D2 (8/13, 62%). Muscular hypotonia was mainly seen with variants in D3 (3/3, 100%) and D2 (5/13, 38%. Contractures were mostly described with variants in D2 (13/13, 100%) and D3 (3/3, 100%). Intellectual disability was mainly observed with variants in both D3 (2/3, 67%), D2 (3/13, 23%), but not in the other recruited domains. Hyporeflexia was detected to a greater extent in D3 (2/3, 67%) and D2 (8/13, 62%).

**Multisystem involvement.** Phenotypes extending to other organ systems were most frequently observed with feeding difficulties (20/22, 91%), leading to failure to thrive in the majority of these patients (n=13). Pulmonary features were reported (17/22, 77%) mostly with pulmonary malformations. Moreover, dysautonomia, feeding difficulties and respiratory abnormalities were present with a high frequency in all patients carrying variants in the extracellular domains. Cardiac features were present in 41% of cases (9/22) and mainly included congenital heart defects. Gastrointestinal features were observed in four patients included hepatomegaly, constipation and gastroesophageal reflux disease. Of note, kidney features were noticed in five patients and included congenital anomalies of the kidney and urinary tract (CAKUT).

**Genetic variants.** One variant was located in the D1 domain (p.Cys65Ser). Two variants were located in the D2 domain, each of these found in individuals of Arabic ethnicity, and including a founder variant (p.Glu219GlyfsTer3) seen in 13 patients with Arabic ancestry. Two variants were located in the D3 domain (p.Lys253Ter; p.Cys270Gly). One variant was located in the D4 domain (p.Ser374ArgfsTer9). Two variants were located in D5 (p.Ser490Ter; c.1438-4A>G). In addition, two variants were located in the D8 domain (c.2497+1G>A; p.Thr815Arg).

**Molecular genetic spectrum in mono- and biallelic IL-6 signaling disorders.** LIFR is composed of 1097 amino acids and contains an extracellular, transmembrane, and cytoplasmic region. The extracellular region includes four cytokine binding domains (D1 CBM1, D2 CBM1, D4 CBM2, D5 CBM2), an immunoglobulin-like domain (D3 Ig-like) and three fibronectin type- III domains (D6 FNIIII, D7 FNIII, D8 FNIII) (1)*.* The *LIFR* cohort consisted of 73 patients of which around 79% had biallelic variants. Seven patients were observed with biallelic variants in D1 CBM1 (n=4 homozygous, n=3 compound heterozygous variants). 22 patients were observed with biallelic variants in D2 CBM1 including the recurrent Arab founder variant in 21 patients (c.653dup, p.Glu219GlyfsTer3). Eleven patients were observed with variants in D3 Ig-like domain (n=8 homozygous, n=4 compound heterozygous). From the eight homozygous patients we observed the recurring variant p.Lys253Ter in seven patients with Slavo-Serbian ethnicities, suggesting this to be a founder variant. None of the variants were present in the health population (gnomAD v3.1.2).

**Neurological features in Stüve-Wiedemann syndrome.** In this context, five *LIFR* patients were reported with reduced fetal movements. 24 *LIFR* patients were reported with hypo/-areflexia, again perhaps explained by the relatively higher expression of *LIFR* in neural tissues. Frequent phenotypes included 24 patients with absent corneal and 17 patellar reflexes. Brain malformations included brain atrophy, brain stem dysgenesis, intracranial hypertension, pontocerebellar hypoplasia, dilated cisterna magna in a group of patients (n=4). Six *LIFR* patients showed acquired brain lesions such as encephalomalacia, multiple FLAIR hyperintensities, and meningitis. Seizures were detailed in nine *LIFR* patients in the early postnatal stage. Three *LIFR* cases were reported with hearing loss. The cohort included cases with sleep disturbance and obstructive sleep apnea (n=5). In the two patients with monoallelic *LIFR* variants in the intracellular region, one patient also had an attention deficit disorder.

**Demographic features.** The patients mainly hailed from Europe (25/74, 34%), the Middle East (38/74, 34%) and Africa (4/74, 5%). Other ethnicities included South Asian (3/74, 4%). and Admixed American (3/74, 4%). Consanguinity was reported for 59% (44/74) in the cohort. Pregnancy and birth were mostly unremarkable without complications. Oligohydramnios was observed in eight *LIFR* patients. The ages of the participants at last follow-up examination were between 4 to 18 years old. While a third of patients died (35%, 26/74), most *LIFR* patients died in infancy (69% from deaths, n=18/26) due to respiratory infections or neonatal hyperthermia that led to cardiorespiratory failure.

**Primary immunodeficiency** was a frequent feature (37/74, 50%), mostly with recurrent infections in the lower and upper respiratory tract (24/74, 32%), skin (4/74, 5%), and eye (17/74, 23%). Eye infections included keratitis and abscesses eventually leading to corneal leukoma in some cases. Less frequent phenotypes included chronic gingivitis (4/74, 5%). Other infections included osteomyelitis (2/74, 3%) and infections with Epstein-Barr virus (EBV) and cytomegalovirus (CMV). Atopic diseases were only described in the recruited cohort (8/74, 11%).

**Skeletal abnormalities** were highly represented (68/74, 92%). These included spine deformities like scoliosis (31/74, 42%), bowing of long bones (61/74, 82%), short extremities (30/74, 41%), camptodactyly (45/74, 61%), and joint abnormalities (31/74, 42%) such as prominent enlarged joints, hyperextensibility, instability (hip/shoulder dislocations) and deformed joints (coxa and genu vara). Feet abnormalities were present less frequently (25/74, 34%). Other phenotypes included susceptibility to fractures (13/74, 18%) and thoracic abnormalities (8/74, 11%) such as pectus excavatum. Short stature was reported frequently with *LIFR* patients (45/74, 61%). Additional radiographic skeletal findings included destructive arthropathy, destructions of femoral heads (6/74, 8%) and abnormal trabeculation (14/74, 19%). On radiographic examinations, *LIFR* patients showed osteoporosis (17/74, 23%), flared metaphyses (32/74, 43%), internal cortical thickening (29/74, 39%) and undertubulation of diaphysis, rarefaction and striation of metaphyses (22/74, 30%).

**Dysautonomia** is a key feature and was observed in 56 *LIFR* patients (56/74, 76%), mostly with body temperature instability (50/74, 68%) including hyperthermia (41/74, 55%), hypothermia (4/74, 5%), intolerance to low temperatures (2/74, 3%) and intolerance to high temperatures (6/74, 8%). Other dysautonomic features included paradoxical sweating (27/74, (36%), mouth ulcerations (18/74, 24%), smooth tongue (20/74, 27%), abnormal pain sensitivity (17/74, 23%), hypersalivation (7/74, 9%), and hypolacrimation (6/74, 8%).

**Additional multisystem features in Stüve-Wiedemann syndrome.** Cardiorespiratory abnormalities were present with high frequency (59/74, 80%). Respiratory distress (42/74, 58%) was mostly found in *LIFR* patients in the neonatal period and led to respiratory failure in some patients (7/74, 9%). The *LIFR* cohort had (13/74, 18%) congenital heart defects including atrial or ventricular septal defects. Pulmonary phenotypes included bronchiectasis and pneumothoraces (3/74, 5%). Less frequent observations included pulmonary hypoplasia, clubbing, and chronic lung diseases.

Dysmorphic craniofacial features were highly present (51/74, 69%). The most common features presented were mouth (28/74, 38%) and dental abnormalities (20/74, 27%), which included mostly pursed lips/mouth (18/74, 24%), high-arched palate (6/74, 8%) and poor dentition with (11/74, 15%). Less frequent features included skull or facial deformities (30/74, 40%), including midface hypoplasia, frontal bossing, craniosynostosis, as well as jaw deformities (22/74, 30%) such as micrognathia (14/74, 19%). Other dysmorphic signs included eyes (18/74, 24%), nose (21/74, 28%) and ear (18/74, 24%) deformities with short palpebral fissures, anteverted nostril, wide nose, and low-set ears. Some other features were short neck (13/74, 18%), milia (6/74, 8%), reduced facial expression/weakness in two *LIFR* patients and a hoarse and nasal voice in one *LIFR* patient. In the *LIFR* cohort, craniofacial dysmorphia manifested in all extracellular variants.

Abdominal features were also found. Feeding difficulties were present in almost all *LIFR* patients (66/74, 89%). Failure to thrive despite adequate caloric intake was observed in 13 *LIFR* patients. Hepatosplenomegaly, pyloric stenosis, and slow motility constipation were observed once. Endocrinological abnormalities were reported with central hypothyroidism (n=1), hypopituitarism, growth hormone deficiency, and hypertrichosis. One *LIFR* case was reported with an absent menarche, whereas another one had early thelarche. Another case was pregnant in the fourth decade of life. One male *LIFR* case with perifascial testicular ectopia. Seven of the *LIFR* patients were seen with single transverse palmar creases and one with no skin dimples on any limb.

In the *LIFR* cohort, eye abnormalities were ubiquitously present in the extracellular region, but almost absent in the three FNIIII domains (D6-D8).

References

1.	Dagoneau N, Scheffer D, Huber C, Al-Gazali LI, Rocco M Di, Godard A, et al. Null Leukemia Inhibitory Factor Receptor (LIFR) Mutations in Stüve-Wiedemann/Schwartz-Jampel Type 2 Syndrome. The American Journal of Human Genetics. 2004 Jun;74(2):298–305. doi:10.1086/381715

2.	Siccha SM, Cueto AM, Parrón‐Pajares M, González‐Morán G, Pacio‐Miguez M, Pozo Á Del, et al. Delineation of the clinical and radiological features of <scp>Stuve–Wiedemann</scp> syndrome childhood survivors, four new cases and review of the literature. Am J Med Genet A. 2021 Jun;185(3):856–65. doi:10.1002/ajmg.a.62010

3.	Jung C, Dagoneau N, Baujat G, Merrer M Le, David A, Rocco M Di, et al. Stüve-Wiedemann syndrome: long-term follow-up and genetic heterogeneity. Clin Genet. 2010 Jun;77(3):266–72. doi:10.1111/j.1399-0004.2009.01314.x

4.	V CD, D G, A M, M LM. New insights in congenital bowing of the femora. Clin Genet. 2004;66(3):169–76.

5.	Gaspar IM, Saldanha T, Cabral P, Vilhena MM, Tuna M, Costa C, et al. Long‐term follow‐up in Stuve–Wiedemann syndrome: A clinical report. Am J Med Genet A. 2008 Jun;146A(13):1748–53. doi:10.1002/ajmg.a.32325

6.	Buonuomo PS, Macchiaiolo M, Cambiaso P, Rana I, Digilio MC, Bartuli A. Long-term follow-up in Stuve–Wiedemann syndrome. Clin Dysmorphol. 2014 Jun;23(2):45–6. doi:10.1097/MCD.0000000000000023

7.	Yeşil G, Lebre AS, Santos S Dos, Güran Ö, Özahi II, Daire VC, et al. Stuve-Wiedemann syndrome: Is it underrecognized? Am J Med Genet A. 2014 Jun;164(9):2200–5. doi:10.1002/ajmg.a.36626

8.	Melone MAB, Pellegrino MJ, Nolano M, Habecker BA, Johansson S, Nathanson NM, et al. Unusual Stüve-Wiedemann syndrome with complete maternal chromosome 5 isodisomy. Ann Clin Transl Neurol. 2014 Jun;1(11):926–32. doi:10.1002/acn3.126

9.	Sarafidis K, Piretzi K, Agakidou E, Kohlhase J, Zafeiriou D. Stüve-Wiedemann syndrome in a neonate. . Pediatrics International. 2015 Jun;57(2):302–4. doi:10.1111/ped.12431

10.	Oxford J. Neuropathies of Stüve-Wiedemann Syndrome due to mutations in leukemia inhibitory factor receptor (LIFR) gene. J Neurol Neuromedicine. 2016 Jun;1(7):37–44. doi:10.29245/2572.942X/2016/7.1068

11.	Alallah J, Alamoudi LO, Makki RM, Shawli A, AlHarbi AT. Stüve-Wiedemann syndrome with a novel mutation in a Saudi infant. Int J Pediatr Adolesc Med. 2022 Jun;9(2):143–6. doi:10.1016/j.ijpam.2021.10.002

12.	Bartik ZI, Sillén U, Djos A, Lindholm A, Fransson S. Whole exome sequencing identifies KIF26B, LIFR and LAMC1 mutations in familial vesicoureteral reflux. PLoS One. 2022 Jun;17(11):e0277524. doi:10.1371/journal.pone.0277524

13.	Palma JA, Yadav R, Gao D, Norcliffe-Kaufmann L, Slaugenhaupt S, Kaufmann H. Expanding the Genotypic Spectrum of Congenital Sensory and Autonomic Neuropathies Using Whole-Exome Sequencing. Neurol Genet. 2021 Jun;7(2). doi:10.1212/NXG.0000000000000568

14.	Schwerd T, Krause F, Twigg SRF, Aschenbrenner D, Chen YH, Borgmeyer U, et al. A variant in IL6ST with a selective IL-11 signaling defect in human and mouse. Bone Res. 2020 Jun;8(1):24. doi:10.1038/s41413-020-0098-z

15.	Cormier-Daire V, Superti-Furga A, Munnich A, Lyonnet S, Rustin P, Delezoide AL, et al. Clinical homogeneity of the St�ve-Wiedemann syndrome and overlap with the Schwartz-Jampel syndrome type 2. Am J Med Genet. 1998 Jun;78(2):146–9. doi:10.1002/(SICI)1096-8628(19980630)78:2<146::AID-AJMG9>3.0.CO;2-M

16.	Rocco M Di, Stella G, Bruno C, Lamba LD, Bado M, Superti-Furga A. Long-term survival in Stuve-Wiedemann syndrome: A neuro-myo-skeletal disorder with manifestations of dysautonomia. Am J Med Genet. 2003 Jun;118A(4):362–8. doi:10.1002/ajmg.a.10242

17.	Superti-Furga A, Tenconi R, Clementi M, Eich G, Steinmann B, Boltshauser E, et al. Schwartz-Jampel syndrome type 2 and St�ve-Wiedemann syndrome: A case for ?Lumping? Am J Med Genet. 1998 Jun;78(2):150–4. doi:10.1002/(SICI)1096-8628(19980630)78:2<150::AID-AJMG10>3.0.CO;2-M

18.	Giedion A, Boltshauser E, Briner J, Eich G, Exner G, Fendel H, et al. Heterogeneity in Schwartz-Jampel chondrodystrophic myotonia. Eur J Pediatr. 1997 Jun;156(3):214–23. doi:10.1007/s004310050587

19.	Kozlowski K, Tenconi R. Stüve-Wiedemann dysplasia in a 3 1/2-year-old boy. Am J Med Genet. 1996 Jun;63(1):17–9. doi:10.1002/(SICI)1096-8628(19960503)63:1<17::AID-AJMG6>3.0.CO;2-P

20.	Al-Gazali LI, Varghese M, Varady E, Talabani J Al, Scorer J, Bakalinova D. Neonatal Schwartz-Jampel syndrome: a common autosomal recessive syndrome in the United Arab Emirates. J Med Genet. 1996 Jun;33(3):203–11. doi:10.1136/jmg.33.3.203

21.	Bayram Y, Karaca E, Akdemir ZC, Yilmaz EO, Tayfun GA, Aydin H, et al. Molecular etiology of arthrogryposis in multiple families of mostly Turkish origin. Journal of Clinical Investigation. 2016 Jun;126(2):762–78. doi:10.1172/JCI84457

22.	Chabrol B, Sigaudy S, Paquis V, Montfort MF, Giudicelli H, Pellissier JF, et al. Stüve-Wiedemann syndrome and defects of the mitochondrial respiratory chain. Am J Med Genet. 1997 Jun;72(2):222–6. doi:10.1002/(SICI)1096-8628(19971017)72:2<222::AID-AJMG18>3.0.CO;2-P

23.	Kosfeld A, Brand F, Weiss AC, Kreuzer M, Goerk M, Martens H, et al. Mutations in the leukemia inhibitory factor receptor (LIFR) gene and Lifr deficiency cause urinary tract malformations. Hum Mol Genet. 2017 Jun;26(9):1716–31. doi:10.1093/hmg/ddx086

24.	Warnier H, Barrea C, Bethlen S, Schrouff I, Harvengt J. Clinical overview and outcome of the Stuve-Wiedemann syndrome: a systematic review. Orphanet J Rare Dis. 2022 Jun;17(1):174. doi:10.1186/s13023-022-02323-8

25.	Chalaris A, Rabe B, Paliga K, Lange H, Laskay T, Fielding CA, et al. Apoptosis is a natural stimulus of IL6R shedding and contributes to the proinflammatory trans-signaling function of neutrophils. Blood. 2007 Jun;110(6):1748–55. doi:10.1182/blood-2007-01-067918

26.	Fischer M, Goldschmitt J, Peschel C, Brakenhoff JPG, Kallen KJ, Wollmer A, et al. A bioactive designer cytokine for human hematopoietic progenitor cell expansion. Nat Biotechnol. 1997 Jun;15(2):142–5. doi:10.1038/nbt0297-142

27.	Berg AF, Ettich J, Weitz HT, Krusche M, Floss DM, Scheller J, et al. Exclusive inhibition of IL-6 trans-signaling by soluble gp130FlyRFc. Cytokine X. 2021 Jun;3(4):100058. doi:10.1016/j.cytox.2021.100058

28.	Baran P, Hansen S, Waetzig GH, Akbarzadeh M, Lamertz L, Huber HJ, et al. The balance of interleukin (IL)-6, IL-6·soluble IL-6 receptor (sIL-6R), and IL-6·sIL-6R·sgp130 complexes allows simultaneous classic and trans-signaling. Journal of Biological Chemistry. 2018 Jun;293(18):6762–75. doi:10.1074/jbc.RA117.001163

29.	Tharyan RG, Annibal A, Schiffer I, Laboy R, Atanassov I, Weber AL, et al. NFYB-1 regulates mitochondrial function and longevity via lysosomal prosaposin. Nat Metab. 2020 Jun;2(5):387–96. doi:10.1038/s42255-020-0200-2

30.	Ge SX, Jung D, Yao R. ShinyGO: a graphical gene-set enrichment tool for animals and plants. Bioinformatics. 2020 Jun;36(8):2628–9. doi:10.1093/bioinformatics/btz931
